# Composite Multi-Stage Transfer Learning with Cross-Scene Adaptation for Personalized In-Vehicle Gaze Estimation

**DOI:** 10.3390/jemr19050119

**Published:** 2026-10-19

**Authors:** Wenlong Dou, Zhonghe Ren, Ziquan Qiu, Fengzhou Fang

**Affiliations:** 1 The International Joint Institute of Tianjin University, Fuzhou, Tianjin University, Tianjin 300072, China; douwenlong@tju.edu.cn; 2 State Key Laboratory of Precision Measuring Technology and Instruments, Laboratory of Micro/Nano Manufacturing Technology (MNMT), Tianjin University, Tianjin 300072, China; qiu2i7uan@tju.edu.cn (Z.Q.); fzfang@tju.edu.cn (F.F.)

**Keywords:** gaze estimation, transfer learning, multi-camera fusion, driver monitoring, cross-scene adaptation

## Abstract

Transferring gaze estimation models from mobile terminals to in-vehicle driving scenarios faces challenges from differences in device form, viewpoint, and imaging conditions. To address this, this study proposes a cross-scene adaptation framework that integrates composite multi-stage transfer learning with distributed multi-camera fusion for personalized in-vehicle gaze estimation. The framework builds upon a multi-level information element fusion baseline (MIEF) and explores three transfer paths (CMP1–CMP3) for progressive personalized adaptation from a large-scale mobile source domain to an in-vehicle target scenario. A distributed multi-camera platform is established, producing gaze calibration data (GCD) for cross-session training and driving gaze and road scene data (DRD) for visualization validation. A Multi-Camera Fusion Model (MCF) is designed with two paradigms, MCF-Lite and MCF-Plus, to balance accuracy improvement and computational complexity. Experimental results demonstrate that the composite multi-stage transfer learning path CMP3 achieves an average error of 0.277 across camera views, with a best single-camera mean error of 0.244. Building on this, the distributed MCF-Plus model with the full C1 + C2 + C3 configuration further reduces the mean error to 0.160 and the standard deviation to 0.127, with 96.88% of predicted gaze points falling within the target calibration block (Error < 0.5). The proposed method provides a feasible technical pathway for personalized adaptation of gaze estimation models from mobile-terminal source domains to in-vehicle multi-camera target scenarios, and offers preliminary experimental evidence for gaze behavior perception and driving gaze heatmap generation in real environments.

## 1. Introduction

Human eye morphology and gaze behavior contain rich information related to emotion and intention [1,2]. Gaze estimation extracts features associated with user gaze behavior and predicts gaze direction or gaze point location using specific computational methods, and has become an important research direction in human–computer interaction [3,4]. With the rapid development of computer vision and the widespread adoption of mobile terminal devices, application scenarios for gaze estimation have expanded from traditional areas such as assistive technologies for people with disabilities, medical diagnosis, and screening to emerging scenarios including smart wearables, mobile terminals, smart homes, and driver assistance and safety applications, demonstrating broad application prospects and important research value [5,6,7,8]. Among these scenarios, in-vehicle driving is particularly important, as driver gaze behavior is closely related to driving safety and human–machine interaction. However, most large-scale gaze datasets are collected in mobile-terminal or laboratory settings, and transferring a gaze estimation model trained on such data to in-vehicle driving scenarios remains challenging due to differences in device form, camera viewpoint, and imaging conditions. Therefore, how to effectively transfer a gaze estimation model from a mobile-terminal source domain to an in-vehicle target scenario has become a key problem.

Current gaze estimation methods can be broadly divided into model-based and appearance-based approaches [1,3]. Appearance-based methods, supported by deep learning, train models using large-scale annotated data and have achieved favorable performance in various scenarios. However, once such methods are trained on a specific scenario dataset, their performance often declines markedly when the application scenario changes, which reveals insufficient cross-scene adaptation capability. In the typical and important application scenario of automotive driving, drivers exhibit a wide range of head poses, hardware configurations are more flexible and can involve multi-camera observations, and environmental illumination is complex and variable [9,10,11,12,13]. These factors impose higher requirements on target-scenario adaptability. Therefore, improving cross-scene adaptation through transfer learning and enhancing prediction accuracy and robustness by exploiting multi-camera observations are key issues addressed in this study. In summary, this study addresses two key problems: first, how to effectively transfer a gaze estimation model from a large-scale mobile-terminal source domain to a personalized in-vehicle target scenario with limited target-domain data; and second, how to exploit multi-camera observations to improve both accuracy and stability under in-vehicle conditions.

To address these issues, this paper proposes a cross-scene gaze estimation adaptation method for personalized driving scenarios and constructs a distributed multi-camera fusion model. The main contributions are as follows:

(1) A composite multi-stage transfer learning framework is proposed for personalized in-vehicle gaze estimation, in which the parameter-inheritance pathway is treated as a design variable and three paths are constructed to evaluate target-only training, source pretraining followed by cross-driver adaptation, and source pretraining followed by same-driver adaptation. This design allows the feature transfer process to be validated under different adaptation settings and makes it possible to isolate the effect of same-driver versus cross-driver adaptation.
(2) A distributed multi-camera fusion strategy is proposed, in which each camera view is independently adapted and the resulting high-level features are fused in a separate stage, so that personalized single-view adaptation and cross-view complementary information can be jointly exploited.
(3) A closed-loop experimental data system is established for validating personalized in-vehicle gaze estimation, covering gaze point calibration, cross-session training, and driving gaze heatmap generation under multiple camera configurations, with two drivers of different characteristics.

The remainder of this paper is organized as follows. Section 2 reviews related work on gaze estimation and transfer learning. Section 3 introduces the multi-level information element fusion baseline model, the composite multi-stage transfer learning method, and the distributed multi-camera fusion model. Section 4 presents the experimental settings, results, analysis, and discussion. Section 5 concludes the paper and discusses future work.

## 2. Related Work

This section reviews related work from two aspects: gaze estimation technology and transfer learning.

### 2.1. Gaze Estimation Technology

Gaze estimation aims to estimate gaze direction or gaze point location from eye images, facial appearance, or related geometric information, and has been widely used in human–computer interaction, intelligent driving, and behavioral analysis [1,3]. Existing methods are mainly categorized as model-based and appearance-based methods. While model-based methods rely on eyeball geometry and features such as the pupil and corneal reflections, providing relatively strong interpretability, appearance-based methods directly learn the mapping between image features and gaze, showing better adaptability in complex scenarios [3,4,5].

In recent years, related studies have mainly focused on geometric modeling, attention mechanisms, multimodal feature fusion, and eye-feature localization [14,15,16,17]. Cheng and Lu [18] introduced Transformer into appearance-based gaze estimation, verifying the effectiveness of self-attention for global feature modeling. Shen et al. [19] utilized Time-of-Flight depth information for 3D gaze estimation, demonstrating the potential of model-based methods under complex imaging conditions. Sinha et al. [20] activated multimodal gaze features using facial landmark heatmaps, thereby improving gaze estimation performance without personal calibration.

Overall, although existing studies have made progress, model-based methods strongly depend on imaging conditions and hardware, whereas appearance-based methods are susceptible to changes in data distribution, device conditions, and application scenarios, leading to performance degradation under cross-scene conditions. This challenge is particularly acute in driving scenarios, where large head movements, complex lighting, and multi-camera observations greatly increase the difficulty of gaze estimation.

### 2.2. Transfer Learning

Transfer learning aims to exploit knowledge from a source domain to improve learning performance in a target-domain task. Its core objective is to mitigate performance degradation caused by limited samples and distribution shifts [21]. In gaze estimation, transfer learning is mainly used to improve model adaptability across datasets, devices, users, and application environments [22,23,24].

In recent years, transfer learning has been preliminarily applied to gaze estimation. Qin et al. [25] proposed a full-face domain-adaptive gaze estimation method based on novel-view synthesis and feature disentanglement, which alleviates performance degradation under cross-domain conditions by expanding the ranges of head pose and gaze and separating gaze-related features. Sun et al. [26] introduced semi-supervised eye landmark detection as an auxiliary task into gaze estimation, achieving knowledge transfer from synthetic to real domains and demonstrating that auxiliary geometric information improves model generalization in real scenarios. Xia et al. [27] addressed domain generalization in cross-domain gaze estimation by proposing a collaborative contrastive learning method, which enhances gaze-related feature representation while suppressing domain-related interference and improving generalization to unseen target domains. These studies have promoted the development of gaze estimation from domain adaptation toward domain generalization.

Several recent studies have further advanced transfer learning for gaze estimation. Solaeman et al. [28] provided a systematic literature review of transfer learning in eye tracking, highlighting that transfer learning is essential for addressing limited labeled data and individual differences. Wang et al. [29] proposed PTGaze, a proxy tuning method for cross-domain gaze estimation that adapts a baseline model to target domains without directly modifying its parameters. Qin et al. [30] introduced UniGaze, which leverages large-scale in-the-wild facial datasets through self-supervised pre-training to improve cross-domain generalization while minimizing reliance on labeled data. Xu et al. [31] analyzed cross-domain gaze estimation from a frequency-domain perspective and proposed an Adaptive Interference Suppression Module (AISM) to suppress sample-specific interfering frequency components. Despite these advances, existing transfer learning methods for gaze estimation share several common limitations. First, most of them perform single-step domain adaptation and do not consider progressive multi-stage parameter inheritance, which is important when the target domain is represented by multiple acquisition sessions. Second, they mainly focus on adaptation issues caused by data-domain differences and individual differences, whereas systematic transfer between different application scenarios remains insufficiently explored, and studies on transferring gaze estimation models from mobile-terminal scenarios to driving scenarios remain limited. Third, they are predominantly designed for single-camera settings, and joint modeling of transfer learning with multi-camera observational information remains relatively underdeveloped [32,33]. In contrast, the proposed method addresses these aspects by combining composite multi-stage transfer learning with distributed multi-camera fusion.

To resolve these issues, this study focuses on cross-scene gaze estimation adaptation under personalized target-domain conditions. Specifically, a small amount of in-vehicle driving scenario data is introduced to adapt a target driver based on a large-scale mobile-terminal source-domain model. Furthermore, considering the characteristics of in-vehicle multi-camera observations, a distributed multi-camera fusion model is constructed to improve gaze point estimation accuracy and stability in the target driving scenario.

Based on the above analysis, this study selects a composite multi-stage transfer learning strategy rather than a single-step domain adaptation approach. The rationale is threefold. First, since the target domain is represented by multiple acquisition sessions collected under different conditions, a single adaptation step is insufficient to capture the progressive distribution shift across sessions, whereas a multi-stage parameter-inheritance pathway can gradually adapt the model across successive target-domain subsets. Second, by constructing three distinct parameter-inheritance paths, the framework makes it possible to isolate the effect of same-driver versus cross-driver adaptation, which is not achievable with a single adaptation scheme. Third, this multi-stage design provides a principled way to exploit source-domain prior knowledge while remaining compatible with the subsequent multi-camera fusion stage, so that personalized single-view adaptation and cross-view complementary information can be jointly exploited.

## 3. Methodology

This section describes the proposed method, including the multi-level information element fusion baseline model, the composite multi-stage transfer learning method, and the distributed multi-camera fusion model.

### 3.1. Multi-Level Information Element Fusion Baseline Model

The baseline model adopted in this study is built upon the Multi-Level Information Element Fusion (MIEF) model established in our previous work [3]. That work proposed a feature fusion method with multi-level information elements to improve appearance-based gaze estimation. The core idea is to organize the input space according to the gaze conduction principle, which explicitly characterizes eye movement, head movement, and their coupling based on human knowledge, rather than relying on a purely data-driven feature extraction pipeline.

The MIEF model constructs a multi-level information element matrix that hierarchically organizes original images, cropped face and eye images, facial and periocular landmarks, pupil localization, head pose, and man-camera distance. Guided by the gaze conduction principle, an input control module selects a task-relevant subset from this matrix to avoid structural redundancy and unnecessary computational overhead. The model then adopts a multi-branch architecture, where image-type inputs are processed through convolutional backbones and geometric-type inputs through fully connected layers; the resulting features are subsequently fused by a fusion module and fed into a regression head to produce two-dimensional gaze point coordinates. This design yields a compact yet extensible baseline that captures complementary facial appearance, binocular appearance, and auxiliary geometric cues—a foundation that not only validates the effectiveness of knowledge-driven input organization but also provides a principled and stable starting point for the subsequent cross-domain transfer and multi-camera fusion stages.

### 3.2. Composite Multi-Stage Transfer Learning Method

Based on the MIEF baseline model proposed in previous work [3], the primary challenge lies in adapting it from the source domain to the target in-vehicle environment. Training a gaze estimation model from scratch using only limited target-domain data tends to yield unstable predictions. Conversely, directly deploying a source-domain model in the target setting often results in significant performance degradation, due to discrepancies in device configuration, camera viewpoint, image appearance, and driver-specific behaviors. To address this issue, a composite multi-stage transfer learning framework built upon MIEF is proposed to investigate how alternative parameter-inheritance pathways facilitate personalized cross-scene adaptation. Its objective is to transfer generic gaze-related representations learned from a large-scale source domain to an in-vehicle multi-view target scenario while maintaining stable gaze point prediction performance across different acquisition sessions.

**Figure 1:**
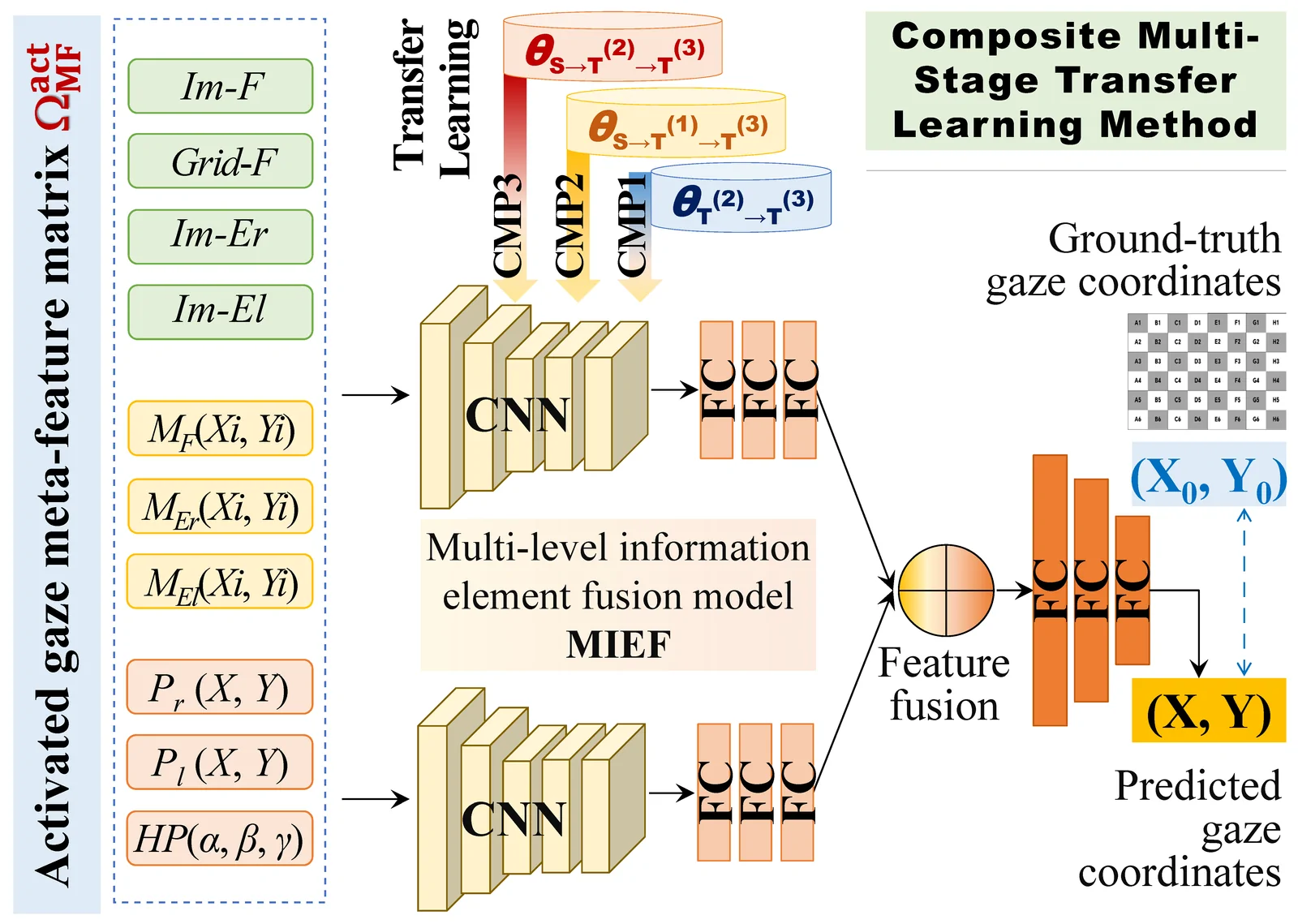
The overview of the proposed composite multi-stage transfer learning framework based on MIEF.

The source-domain pretraining dataset is denoted as 𝒟_S_ and the complete target-domain data collection as 𝒟_T_. Specifically, 𝒟_T_ is partitioned into three subsets, 𝒟_T_^(1)^, 𝒟_T_^(2)^, and 𝒟_T_^(3)^. Among these, 𝒟_T_^(2)^ and 𝒟_T_^(3)^ are both collected from the same driver, whereas 𝒟_T_^(1)^ is acquired from a different driver. An overview of the proposed composite multi-stage transfer learning framework based on MIEF is provided in Figure 1.

Each target-domain input sample from 𝒟_T_ is transformed into the activated gaze meta-feature matrix Ω_MF_^act^, following the meta-feature construction strategy from previous work [3]. This representation integrates face images, eye-region images, and geometric cues, which are then processed by the MIEF model for feature extraction, multimodal feature fusion, and two-dimensional gaze-coordinate regression. During supervised training, the predicted gaze coordinates (*X*, *Y*) are optimized against the ground-truth coordinates (*X*_0_, *Y*_0_) by minimizing the coordinate-regression loss *L* defined in Equation (1).

(1) $$L=\frac{1}{2}[{\left(X-X_{0}\right)}^{2}+{\left(Y-Y_{0}\right)}^{2}]$$

To evaluate the impact of different parameter-inheritance strategies, a Composite Multi-Stage Transfer Learning Path (CMP) is introduced. Based on this framework, three specific paths, denoted as CMP1, CMP2, and CMP3, are designed. All three paths share the same MIEF backbone but differ in parameter initialization and the choice of target-domain adaptation subsets.

In CMP1, the model is trained solely on the target-domain adaptation sets 𝒟_T_^(2)^ and 𝒟_T_^(3)^, yielding the parameter set *θ*_T_^(2)^_→T_^(3)^. This mode does not involve source-domain pretraining and thus serves as a target-only baseline, indicating the level of performance that can be achieved using only limited target-domain samples.

In CMP2, the model is first pretrained on the source-domain dataset 𝒟_S_, yielding the source-domain parameter set *θ*_S_. Subsequently, it undergoes a composite transfer learning process consisting of two sequential adaptation steps on the training partitions of 𝒟_T_^(1)^ and 𝒟_T_^(3)^. The resulting parameter set, denoted as *θ*_S→T_^(1)^_→T_^(3)^, characterizes the adaptation pathway from the source domain through the two composite transfer stages.

In CMP3, the model is first pretrained on the source-domain dataset 𝒟_S_, yielding the source-domain parameter set *θ*_S_. Subsequently, it undergoes a composite transfer learning process consisting of two sequential adaptation steps on the training partitions of 𝒟_T_^(2)^ and 𝒟_T_^(3)^. The resulting parameter set, denoted as *θ*_S→T_^(2)^_→T_^(3)^, characterizes the adaptation pathway from the source domain through the two composite transfer stages. It is worth noting that 𝒟_T_^(1)^ is collected from a different driver than 𝒟_T_^(2)^ and 𝒟_T_^(3)^, making CMP2 a cross-driver adaptation path. In contrast, CMP3 maintains driver consistency from the first transfer step onward, as both 𝒟_T_^(2)^ and 𝒟_T_^(3)^ originate from the same driver.

Through these three parameter-inheritance paths, the proposed framework evaluates the respective effects of target-only training, source pretraining followed by cross-driver target-domain adaptation, and source pretraining followed by same-driver target-domain adaptation. The specific datasets corresponding to 𝒟_S_, 𝒟_T_^(1)^, 𝒟_T_^(2)^, and 𝒟_T_^(3)^ are introduced in Section 4.1.2.

### 3.3. Distributed Multi-Camera Fusion Strategy

**Figure 2:**
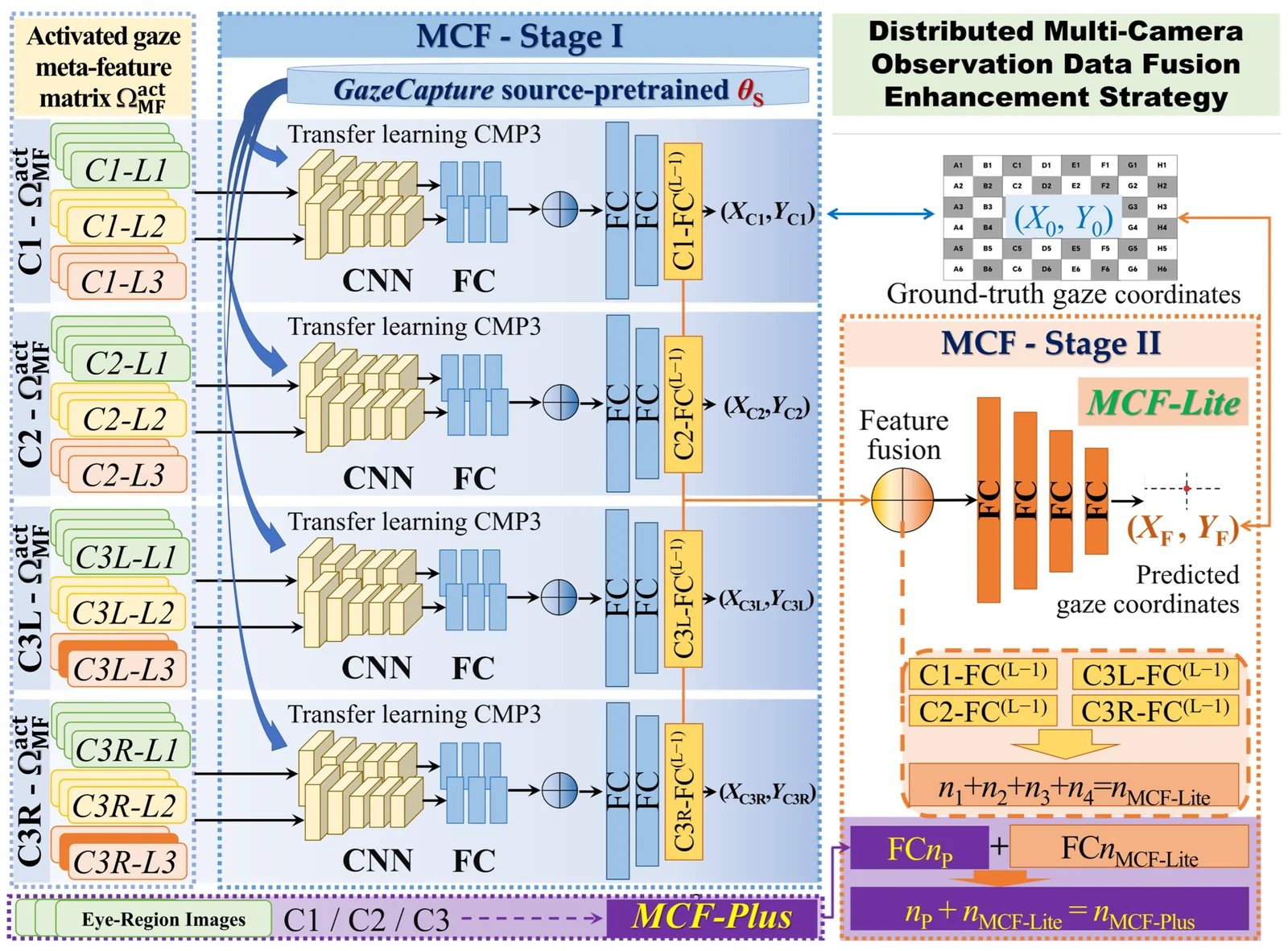
The overview of the proposed distributed multi-camera observation data fusion enhancement strategy.

A distributed multi-camera observation fusion strategy, termed Multi-Camera Fusion (MCF), is proposed to address the vulnerability of single-camera gaze estimation to variations in viewpoint, visible facial regions, and head pose. This strategy employs the single-camera MIEF models as camera-specific feature extractors and fuses high-level features computed from temporally synchronized observations. Through this integration, complementary information from multiple viewpoints can be jointly modeled, thereby enhancing both the accuracy and stability of gaze point estimation. Within the in-vehicle setting, three camera configurations are examined: C1 + C2, C1 + C3, and C1 + C2 + C3. Here, C1 and C2 denote monocular camera units, while C3 represents a binocular camera unit that supplies the C3L and C3R view streams for branch-level modeling and feature fusion. An overview of the proposed distributed multi-camera observation data fusion enhancement strategy is illustrated in Figure 2.

In Stage I, the synchronized samples from each camera view are independently processed in parallel by a dedicated MIEF branch. The activated gaze meta-feature matrix Ω_MF_^act^ is organized into three level-wise components. For example, the C1 branch receives C1-L1, C1-L2, and C1-L3. The C2, C3L, and C3R branches follow the same three-level organization. Each branch is initialized with the GazeCapture source-pretrained parameter set *θ*_S_ and is then fine-tuned on the corresponding target-domain adaptation data, following the CMP3 transfer learning path. The final fully connected layer, FC^(L)^, produces a stream-specific gaze-coordinate prediction, for example (*X*_C1_, *Y*_C1_), which is supervised against the ground-truth coordinates (*X*_0_, *Y*_0_) using the coordinate-regression loss. The output of the penultimate fully connected layer, FC^(L−1)^, is retained as a camera-specific high-level feature of dimension *n*_1_ for Stage II fusion.

In Stage II, the parameters of each camera-specific MIEF branch are frozen, and the branches serve as fixed feature extractors. For each temporally synchronized sample, the outputs of the FC^(L−1)^ layers are concatenated and passed to a fusion regression network that produces the final fused gaze coordinates (*X*_F_, *Y*_F_). Specifically, the C1 + C2 configuration concatenates the outputs from the C1 and C2 branches, the C1 + C3 configuration concatenates the outputs from the C1, C3L, and C3R branches, and the C1 + C2 + C3 configuration concatenates the outputs from all four available branches. Therefore, although C1 + C3 involves two physical camera units, it forms a three-stream fusion architecture at the network level because C3 provides separate left- and right-view feature streams. The fusion regression network is optimized using the loss *L*_F_ defined in Equation (2), while the parameters of all Stage I branches remain fixed.

(2) $$L_{F}=\frac{1}{2}[{\left(X_{F}-X_{0}\right)}^{2}+{\left(Y_{F}-Y_{0}\right)}^{2}]$$

Based on the complexity of the Stage II process, two fusion paradigms are designed: MCF-Lite and MCF-Plus. MCF-Lite employs only the concatenated high-level features extracted from the frozen MIEF branches and trains a lightweight fusion regression network. MCF-Plus retains this multi-camera representation and augments it with an auxiliary image-level feature, which is extracted by a convolutional neural network branch with fully connected layers from the synchronized eye-region images corresponding to the selected camera configuration. In this way, MCF-Plus complements, rather than replaces, the camera-specific high-level features.

#### 3.3.1. C1 + C2 (Dual Monocular Cameras)

In the C1 + C2 configuration, each monocular stream provides a three-level activated gaze meta-feature matrix, specifically C1-L1, C1-L2, and C1-L3 for C1, and C2-L1, C2-L2, and C2-L3 for C2. The two streams are processed by independent MIEF branches, both initialized with the GazeCapture source-pretrained parameter set *θ*_S_ and fine-tuned via the CMP3 transfer learning path. During Stage I, the FC^(L)^ output of each branch yields a stream-specific gaze prediction, denoted as (*X*_C1_, *Y*_C1_) and (*X*_C2_, *Y*_C2_), respectively, which are supervised against the ground-truth coordinates (*X*_0_, *Y*_0_) through branch-specific coordinate-regression losses. These intermediate predictions serve solely to optimize the two view-specific branches. Upon completion of Stage I, the C1 and C2 branches are frozen, and the outputs of their FC^(L−1)^ layers are passed to Stage II for fusion. Specifically, MCF-Lite concatenates these two 720-dimensional feature vectors into a 1440-dimensional fusion representation. MCF-Plus further augments this representation by extracting an additional 256-dimensional feature from the synchronized C1 + C2 eye-region images. Concatenating this auxiliary feature with the 1440-dimensional MCF-Lite representation yields a 1696-dimensional input to the final fusion regressor.

#### 3.3.2. C1 + C3 (Monocular + Binocular Camera)

In the C1 + C3 configuration, although this setup involves only two physical camera units, C3 is binocular and is therefore decomposed into two separate view streams, C3L and C3R, for branch-level processing. Stage I comprises three independently adapted MIEF branches, namely C1, C3L, and C3R. Each branch receives its own three-level input set, produces a view-specific gaze-coordinate prediction for coordinate-regression supervision, and, after the CMP3-adapted parameters are frozen, supplies a 720-dimensional output from its FC^(L−1)^ layer. In Stage II, MCF-Lite concatenates the FC^(L−1)^ outputs from the three branches to form a 2160-dimensional fusion representation. MCF-Plus further augments this representation by extracting a 384-dimensional auxiliary feature from the synchronized C1 + C3 eye-region images. Concatenating this feature with the 2160-dimensional MCF-Lite representation yields a 2544-dimensional input to the final fusion regressor. A key characteristic of this configuration is that the two views of C3 are maintained as separate feature streams until the final fusion stage, thereby preserving binocular information for later integration.

#### 3.3.3. C1 + C2 + C3 (Full Multi-Camera Setup)

In the C1 + C2 + C3 configuration, Stage I encompasses four camera-specific streams, namely the two monocular streams C1 and C2, together with the C3L and C3R streams derived from the binocular camera C3. For each stream, the three-level activated meta-features are processed by a dedicated MIEF branch, each initialized with the same source-domain parameter set *θ*_S_ and fine-tuned via the CMP3 transfer learning path. During Stage I, the FC^(L)^ outputs of these branches provide stream-specific coordinate predictions for supervision, while the FC^(L−1)^ outputs are retained after branch freezing to serve as feature-level inputs for Stage II. Accordingly, MCF-Lite concatenates the four 720-dimensional FC^(L−1)^ outputs from C1, C2, C3L, and C3R to construct a 2880-dimensional representation for the Stage II regression network. MCF-Plus further augments this representation by extracting a 512-dimensional auxiliary feature from the synchronized eye-region images of the full C1 + C2 + C3 setup. Concatenating this auxiliary feature with the 2880-dimensional MCF-Lite representation yields a 3392-dimensional input to the final fusion regressor. Among the three configurations, this scheme delivers the most comprehensive Stage II representation.

In summary, the proposed MCF model consists of camera-specific MIEF branches adapted via the CMP3 path in Stage I, a feature-level fusion stage in Stage II that concatenates the adapted high-level features and feeds them into a fusion regression network, and two fusion paradigms (MCF-Lite and MCF-Plus) that differ in whether an auxiliary image-level feature is additionally incorporated. For each camera configuration, the number of parallel branches is determined by the number of available view streams: two for C1 + C2, three for C1 + C3 (including C3L and C3R), and four for C1 + C2 + C3. This design allows personalized single-view adaptation and cross-view complementary information to be jointly exploited within a unified framework.

## 4. Experiments and Discussion

This section presents the experimental results and discussion of the proposed composite multi-stage transfer learning framework and multi-camera fusion strategy, covering dataset construction, experimental settings, performance evaluation of parameter-inheritance paths, multi-camera fusion performance, computational efficiency, and driving gaze heatmap application.

### 4.1. Datasets and Experimental Settings

#### 4.1.1. Data Acquisition Platform

**Figure 3:**
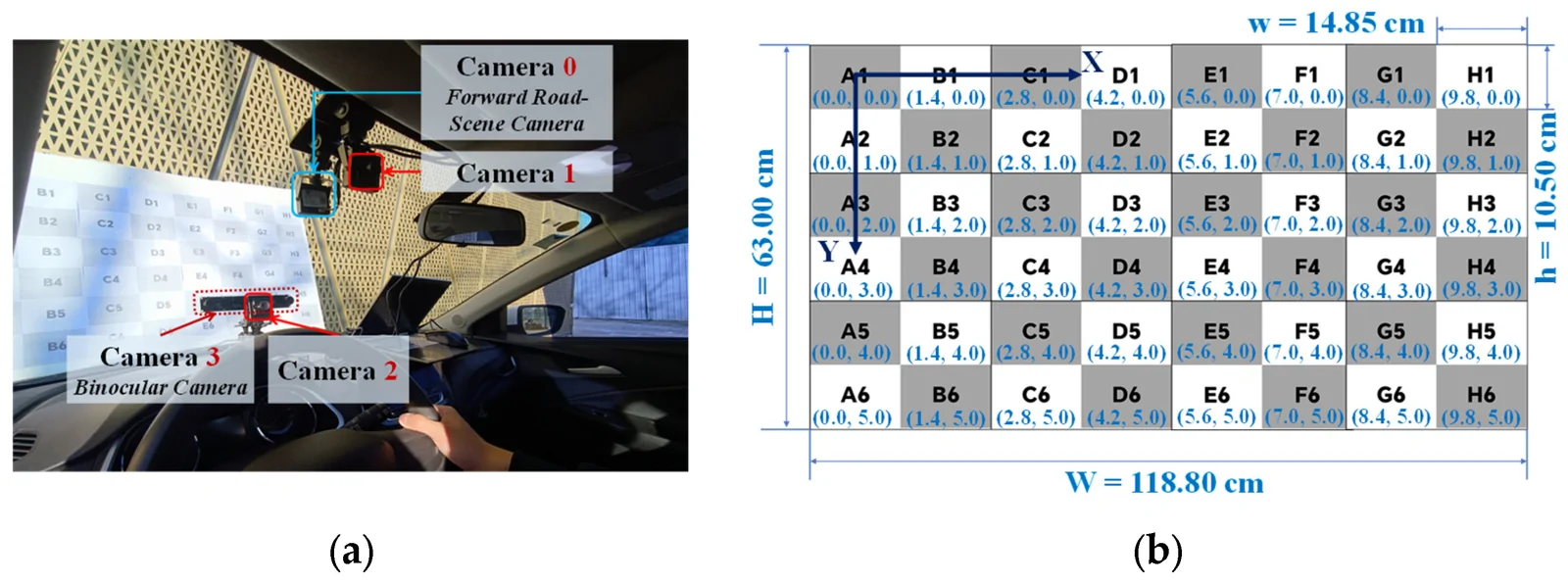
Overview of the data acquisition platform. (**a**) In-vehicle multi-camera setup and driving scene layout. (**b**) Gaze point calibration board with 8 × 6 grid blocks indexed from A1 to H6.

The overall data acquisition platform is illustrated in Figure 3, where Figure 3a shows the in-vehicle multi-camera setup and driving scene layout, and Figure 3b depicts the gaze point calibration board.

To obtain ground-truth gaze labels, the calibration board was placed within the forward field of view of the driver, as shown in Figure 3b. The board measures 118.80 cm × 63.00 cm and is arranged in an 8 × 6 grid, yielding 48 gaze point blocks, each measuring 14.85 cm × 10.50 cm and indexed from A1 to H6. During calibration, the subject sequentially fixates on each block, while multiple cameras synchronously capture the facial and eye images. This procedure establishes the correspondence between each recorded sample and its corresponding ground-truth gaze region.

#### 4.1.2. Dataset Construction

Based on the above platform, a series of datasets were collected, including Gaze Calibration Data (GCD) and Driving Gaze and Road Scene Data (DRD). In addition, the public GazeCapture dataset [34] was adopted as the source-domain pretraining dataset in the transfer-learning experiments. Following the notation introduced in Section 3.2, 𝒟_S_ denotes the source-domain pretraining dataset, instantiated by GazeCapture, while 𝒟_T_ denotes the complete target-domain GCD collection. Specifically, GCD1, GCD2, and GCD3 instantiate 𝒟_T_^(1)^, 𝒟_T_^(2)^, and 𝒟_T_^(3)^, respectively. Each of these GCD subsets contains 11,520 data frames, of which 9216 are allocated for training and 2304 for testing. The DRD dataset was collected in this study using the same in-vehicle multi-camera platform described above and is not a publicly available dataset. It is used solely to validate gaze mapping and heatmap visualization in real-world driving scenarios.

For each GCD subset, the training and testing sets were split by acquisition round rather than by frame-level random sampling. During data acquisition, a custom acquisition control software was used to guide the participant to fixate on each gaze region in a predefined order. One acquisition round refers to a complete traversal from the first predefined gaze region to the last predefined gaze region. Multiple such rounds were collected for each GCD subset. The majority of the rounds were used for the training set, while one randomly selected round was held out as the testing set. Since the training and testing sets are drawn from different acquisition rounds, they do not share any overlapping or temporally adjacent frames. This round-wise splitting strategy avoids data leakage and strong temporal correlation between the training and testing sets, ensuring that the reported performance reflects the model’s ability to generalize to unseen, independently acquired frames.

The target-domain data were collected from two participants in total. Driver A (male, 30 years old, wearing glasses, height 175 cm) contributed GCD1, which instantiates 𝒟_T_^(1)^ and was used as the cross-driver adaptation subset. Driver B (male, 31 years old, not wearing glasses, height 177 cm) contributed GCD2 and GCD3, which instantiate 𝒟_T_^(2)^ and 𝒟_T_^(3)^, respectively, and were acquired in two separate sessions. It should be noted that GCD2 and GCD3 originate from the same driver and thus are not independent of each other; accordingly, they should not be treated as independent observations in the evaluation. The rationale for assigning GCD2 and GCD3 to the same driver is closely related to the design of the parameter-inheritance paths. As described in Section 3.2, the proposed framework evaluates three adaptation settings through CMP1, CMP2, and CMP3: target-only training, source pretraining followed by cross-driver target-domain adaptation, and source pretraining followed by same-driver target-domain adaptation. Specifically, CMP2 uses GCD1 from Driver A as the cross-driver adaptation subset, whereas CMP3 uses GCD2 and GCD3 from Driver B as the same-driver adaptation subsets. Therefore, GCD2 and GCD3 were deliberately collected from the same driver in order to construct the same-driver adaptation path CMP3 and to isolate the effect of same-driver versus cross-driver adaptation. This design choice was intended to validate the feature transfer process of the proposed method under different adaptation settings, rather than to claim generalizability across the driver population. The implications of the limited participant pool are further discussed in Section 4.6.

#### 4.1.3. Experimental Environment and Training Parameters

The experimental environment and training parameters are summarized in Table 1. These hyperparameters were selected empirically to balance training stability and convergence speed.

### 4.2. Evaluation of Composite Multi-Stage Transfer Learning Paths

A series of experiments was conducted on GCD1, GCD2, and GCD3 to evaluate the performance of parameter-inheritance paths for cross-scene adaptation.

Experiment 1 was carried out on GCD1 under three training modes. The first mode, denoted as Direct Source Testing (DST), directly evaluates the source-pretrained model on the target-domain data without any transfer learning. The second mode, termed Target-Only Training (TOT), trains the model from scratch using only the limited target-domain samples. The third mode, referred to as Source-to-Target Transfer (STT), initializes the model with source-pretrained parameters and fine-tunes it on the target-domain adaptation set.

Experiment 2 was conducted on GCD2, retaining the TOT and STT modes for comparison, while DST was excluded due to its consistently inferior performance observed in Experiment 1.

Experiment 3 was designed to validate the performance of the proposed composite multi-stage transfer learning framework under progressive cross-scene adaptation settings. This experiment jointly leverages GCD1, GCD2, and GCD3, and adopts three distinct composite multi-stage transfer learning paths—denoted as CMP1, CMP2, and CMP3—as defined in Section 3.2. These paths correspond to different parameter-inheritance pathways that progressively transfer knowledge across successive target-domain subsets.

Table 2 summarizes the prediction error statistics of Experiment 1, Experiment 2, and Experiment 3. In each experiment, independent gaze estimation evaluations were conducted on different camera views, including C1, C2, and the left and right views of C3 (denoted as C3L and C3R). For each gaze prediction evaluation, different training modes defined in previous sections were adopted, varying in whether source-domain pretraining was used, the target-domain adaptation path for transfer learning, and the evaluation dataset.

In terms of performance assessment, the mean and standard deviation of the Euclidean gaze point prediction error, denoted as *μ (E*) and *σ (E*), respectively, serve as the primary evaluation metrics, characterizing the central tendency and dispersion of the prediction errors. In addition, two supplementary metrics are defined: the percentage of test samples with Error (*E*) < 0.5, whose predicted gaze points fall within the corresponding target block on the calibration board; and the percentage of test samples with Error (*E*) > 1.5, whose predicted gaze points deviate substantially from the corresponding target block. It should be noted that the Error (*E*) > 1.5 metric represents the proportion of test samples whose predicted gaze points deviate substantially from the corresponding target block. As the model accuracy improves through composite multi-stage transfer learning and multi-camera fusion, the proportion of such severely deviated samples decreases accordingly. Therefore, a value of 0.00 in this column indicates that, under the corresponding configuration, no test sample exhibited a severe deviation from the target block, rather than indicating zero error.

To provide an indication of the robustness of the reported mean errors, approximate 95% confidence intervals (CI) are computed from the mean and standard deviation of the prediction errors, as defined in Equation (3):

(3) $$\mathrm{CI}=\mu \left(E\right)\pm 1.96\cdot \frac{\sigma \left(E\right)}{\sqrt{n}}$$

where *μ (E*) and *σ (E*) denote the mean and standard deviation of the prediction errors, respectively, and *n* = 2304 is the number of test frames. Given the large sample size, the sampling distribution of the mean error is approximately normal by the central limit theorem, and this normal approximation therefore provides a reasonable estimate of the confidence interval. The resulting CI is expected to be narrow and to not overlap substantially between the compared configurations, which would indicate that the observed differences are unlikely to be caused by random variation.

In Experiments 1 and 2, the results showed that directly applying the source-domain model to the target driving scenario (DST) produced large prediction errors, indicating that cross-scene data distribution differences substantially weakened the effectiveness of appearance-based gaze estimation models. When only a small number of target-domain samples were used for target-only training (TOT), the model could complete basic fitting, but its prediction stability under cross-session conditions remained limited. By contrast, transfer-learning-based training (STT) substantially reduced prediction errors, as demonstrated in Experiment 1 and Experiment 2 on GCD1 and GCD2, respectively. For instance, on camera view C1 in Experiment 1, STT reduced the mean error from 1.952 to 0.374, significantly outperforming DST, for which the mean error was 7.023. On camera view C1 in Experiment 2, STT reduced the standard deviation from 3.929 to 0.242. These results indicate that introducing source-domain prior knowledge and updating parameters with target-domain samples is an effective way to achieve personalized cross-scene adaptation for in-vehicle gaze estimation models.

In Experiment 3, among the three parameter-inheritance paths evaluated, CMP3 achieved the best overall performance. CMP1 consistently produced the highest errors across all camera configurations, whereas CMP2 and CMP3 substantially reduced the prediction errors, with CMP3 achieving the lowest error for every evaluated configuration. This result indicates that transfer effectiveness depends not only on source-domain pretraining but also on the relevance of the target-domain data selected for adaptation. Compared with CMP1 and CMP2, CMP3 used target-domain data acquired closer to the final cross-session testing condition and yielded more stable prediction performance. These findings supported the use of multiple independently constructed parameter-inheritance paths for personalized cross-scene adaptation, as the progressive transfer strategy effectively mitigated domain discrepancies across successive target-domain subsets.

The approximate 95% CI further supports this observation. For example, on camera view C1, the mean error of CMP3 is 0.266 with a 95% CI of [0.260, 0.272], which is clearly separated from that of CMP1 (0.969, 95% CI [0.945, 0.993]) and CMP2 (0.313, 95% CI [0.306, 0.320]). Similar separation is observed across the other camera views, indicating that the improvements achieved by CMP3 are consistent and unlikely to be attributable to random variation.

### 4.3. Performance Evaluation of Multi-Camera Fusion

Building upon the composite multi-stage transfer learning path CMP3, further experiments were conducted to validate the multi-camera observation data fusion enhancement strategy. The Relative Error Reduction (%) column reports the percentage reduction in mean error relative to the single-camera baseline. The experimental results are presented in Table 3.

Table 3 compares the performance of the single-camera baseline and multi-camera fusion configurations. The single-camera baseline is established as the average performance across the four individual camera views (C1, C2, C3L, and C3R), each evaluated under the CMP3 transfer learning path. This baseline achieves a mean error *μ (E*) of 0.277 with a standard deviation *σ* (*E*) of 0.155. Furthermore, as can be observed from the per-view results in Table 2, single-camera gaze estimation suffers from notable viewpoint-dependent performance variations and limited stability. In contrast, all multi-camera fusion configurations substantially reduce both the mean error and standard deviation. Specifically, the C1 + C2 configuration lowers the mean error to approximately 0.185 (MCF-Lite), representing a relative reduction of 30.5% compared to C1 (CMP3) and 43.6% compared to C2 (CMP3). The C1 + C3 configuration further reduces the mean error to 0.166 with MCF-Plus (and 0.169 with MCF-Lite), while the full C1 + C2 + C3 setup achieves the best performance, with mean errors of 0.162 (MCF-Lite) and 0.160 (MCF-Plus). In terms of the supplementary metric Error (*E*) < 0.5, the single-camera baseline achieves an average of 91.67%, whereas multi-camera fusion configurations consistently exceed 95%, with the full-camera setup reaching 96.88% for MCF-Plus. Overall, the results demonstrate that multi-camera fusion not only significantly improves gaze estimation accuracy but also enhances prediction stability, with the full C1 + C2 + C3 configuration delivering the most substantial gains.

It should be noted that the difference between MCF-Lite and MCF-Plus is small in the C1 + C2 + C3 configuration, where the mean error decreases only from 0.162 to 0.160. The approximate 95% CIs of these two configurations are [0.157, 0.167] and [0.155, 0.165], respectively, which overlap substantially. Therefore, the difference between MCF-Lite and MCF-Plus in this configuration should not be interpreted as a clear advantage of the more complex fusion strategy. In contrast, the advantage of MCF-Plus over the single-camera baseline, and the advantage of multi-camera fusion over single-camera estimation, are supported by clearly separated CI and are therefore more robust. The role of MCF-Plus is therefore better understood as providing a modest and consistent refinement rather than a substantial improvement over MCF-Lite.

To assess the competitiveness of the proposed method relative to representative existing strategies, two baseline categories are considered. The first is a transfer-learning-based gaze estimation baseline, which follows the single-step domain adaptation strategy commonly adopted in cross-domain gaze estimation and corresponds to the STT mode in Table 2. As shown in Table 2, on camera view C1, the single-step transfer baseline achieves a mean error of 0.374, whereas the proposed CMP3 path reduces the mean error to 0.266. The second is the single-camera baseline, which is established as the average performance across the four individual camera views under the CMP3 transfer learning path, as reported in Table 3. The single-camera baseline achieves a mean error of 0.277, whereas the proposed MCF-Plus model with the full C1 + C2 + C3 configuration reduces the mean error to 0.160. These comparisons indicate that the proposed composite multi-stage transfer learning and distributed multi-camera fusion strategies consistently outperform the corresponding representative baselines within the evaluated setup.

To ensure that the observed improvement is attributable to the proposed fusion mechanism rather than merely to the use of more cameras or more training data, the training data volume was kept consistent between the single-camera models and the multi-camera fusion models. Specifically, the single-camera baseline is established as the average performance across the four individual camera views (C1, C2, C3L, and C3R) under the same CMP3 transfer learning path, using the same amount of target-domain training data as the multi-camera fusion configurations. Therefore, the gain achieved by the multi-camera fusion configurations cannot be explained by an increase in training data volume, and is more directly attributable to the proposed two-stage adaptation-and-fusion mechanism. The single-camera baseline reported in Table 3 represents the performance achieved without any cross-view fusion, and therefore serves as a baseline for comparison. This baseline achieves a mean error of 0.277 with a standard deviation of 0.155. As shown in Table 2, the per-view predictions differ substantially across camera views: under the CMP3 path, the mean errors on C1, C2, C3L, and C3R are 0.266, 0.328, 0.268, and 0.244, respectively. In contrast, the proposed MCF-Plus model reduces the mean error to 0.160, which is substantially lower than any individual view and also lower than the single-camera average. This gap indicates that the improvement cannot be explained by simply using more cameras, and that the learned fusion mechanism plays a substantial role.

The complementarity among cameras installed at different positions is reflected in three aspects: observation viewpoints, visible facial regions, and geometric constraint information. The experimental results demonstrate that the MCF model effectively integrates high-level multi-camera representations, leading to consistent improvements in both gaze point prediction accuracy and stability. Among the evaluated fusion configurations, those incorporating binocular observation information consistently outperform their monocular counterparts. This advantage can be attributed to the richer head-pose and local appearance cues provided by binocular inputs, which are particularly beneficial for gaze estimation under complex driving conditions with significant head motion and viewpoint variations. The full C1 + C2 + C3 configuration achieves the best overall performance, further confirming that increasing the number and diversity of observation sources enables more comprehensive multi-view collaborative modeling and greater exploitation of cross-camera complementary information.

The approximate 95% CI further confirms the robustness of these improvements. The single-camera baseline achieves a mean error of 0.277 with a 95% CI of [0.271, 0.283], whereas the full C1 + C2 + C3 configuration with MCF-Plus achieves a mean error of 0.160 with a 95% CI of [0.155, 0.165]. The CIs of the compared configurations do not overlap substantially, indicating that the observed accuracy gains from multi-camera fusion are consistent rather than attributable to random variation.

### 4.4. Computational Efficiency Evaluation

The runtime on the experimental platform was used as the metric to compare the computational cost of the single-camera model and the MCF-Plus model. All measurements were obtained on the same platform, so the comparison is consistent and directly comparable. For the single-camera model, the training time per batch was 0.171 s, the average inference time was 0.041 s per sample, and the processing speed reached approximately 24 frames per second. After adopting the MCF-Plus model with the full C1 + C2 + C3 configuration, the training time per batch increased to 0.228 s, and the average inference time rose to 0.065 s per sample, corresponding to approximately 15 frames per second. This overhead is mainly caused by the parallel processing of multiple camera streams in Stage I and the additional fusion regression network in Stage II. In terms of the targeted precision, MCF-Plus reduces the mean error from 0.277 to 0.160, while the inference speed remains sufficient for real-time applications. Therefore, the integration of multiple camera views provides a favorable trade-off between accuracy and computational cost. Future work will focus on model compression and lightweight design for deployment-oriented applications, including a complete analysis of model parameters and FLOPs and further optimization of the fusion architecture.

### 4.5. Driving Gaze Heatmap Application

**Figure 4:**
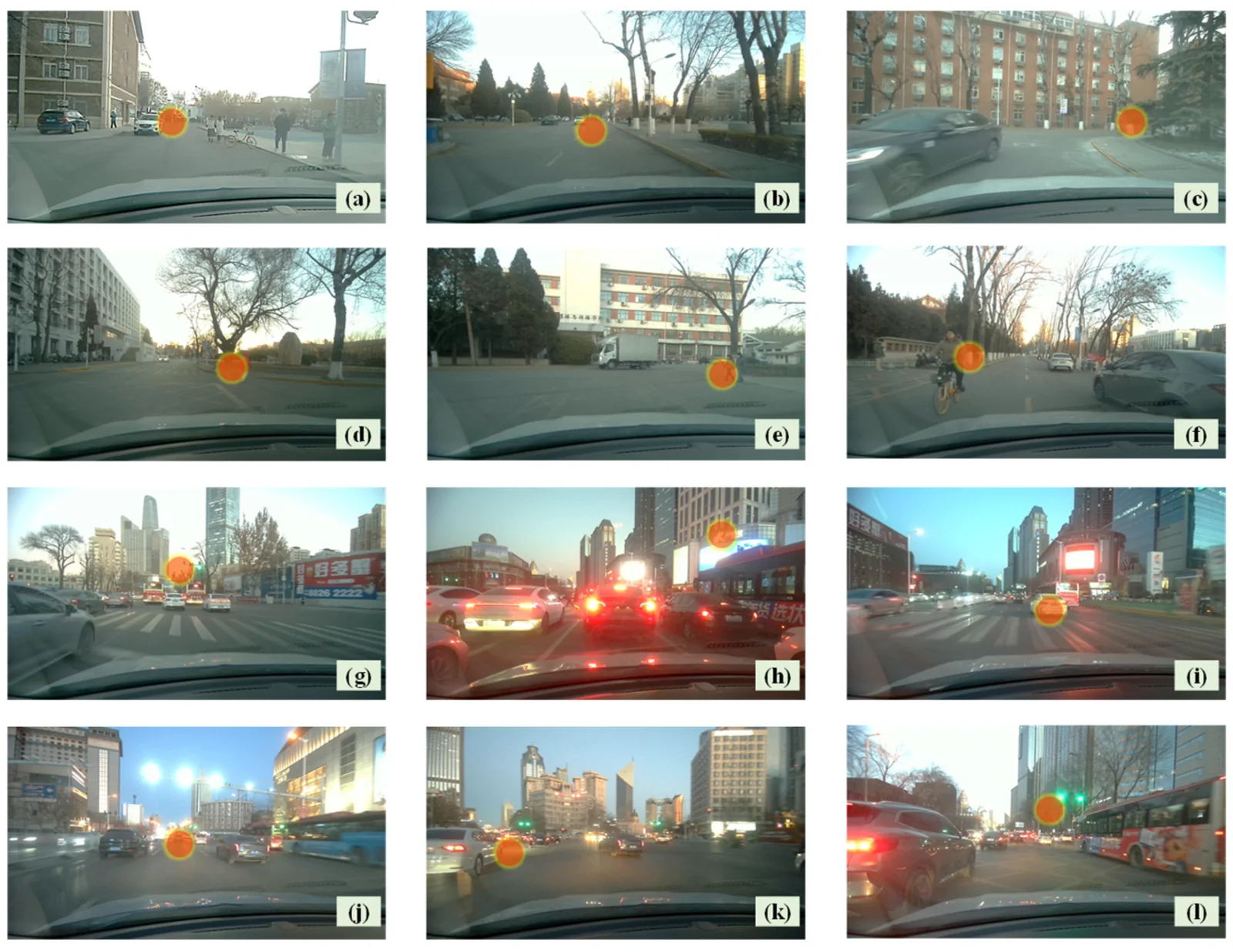
Schematic examples of driving gaze heatmaps projected onto forward road-view images from the DRD dataset. (**a**–**l**) Different road scenes from the DRD dataset.

As a demonstration example, based on the multi-camera synchronized images from the DRD dataset, the trained MCF-Plus model with the C1 + C2 + C3 configuration was employed to infer the gaze point coordinates of the driver at each sampling instant. Combined with the geometric calibration of the road-scene camera, the gaze point coordinates were mapped onto the forward road image plane via perspective projection transformation, thereby generating driving gaze heatmaps, as illustrated in Figure 4. This application example established a complete closed loop from eye observation to gaze point prediction and road-space mapping, providing a practical demonstration for visualizing and functionally applying gaze estimation results in real driving scenarios. The 12 samples shown in Figure 4 cover a variety of driving scenes with different visual conditions, so that the gaze distribution can be examined under diverse scenarios.

As shown in Figure 4, the generated heatmaps effectively captured the spatial aggregation characteristics of gaze distribution in response to changing road scenes during driving. Compared with discrete gaze point coordinates, heatmap visualization offered a more intuitive representation of how drivers allocated visual attention to critical forward regions in conjunction with the visual scene characteristics, thereby providing a more continuous and interpretable basis for driving behavior analysis.

Furthermore, beyond visualization and application demonstration, driving gaze heatmaps could be leveraged for driving behavior assessment and intelligent driver-vehicle human–machine collaboration analysis. By statistically analyzing gaze distribution, stability, and region-switching patterns under varying road conditions, quantitative evaluation metrics for driving safety could be derived. These metrics, in turn, could inform interface optimization, risk-warning strategy design, and road-scene understanding in advanced driver assistance systems. It should be noted that the driving gaze heatmap presented here serves as a demonstration example rather than a full comparative evaluation. A systematic comparison of different gaze estimation approaches on the DRD dataset will be conducted in future work.

### 4.6. Limitations

Although the proposed framework achieves promising results within the evaluated setup, several limitations should be acknowledged.

It should be emphasized that the target-domain evaluation in this study is based on only two participants, and that GCD2 and GCD3 were collected from the same driver across two sessions. Although each GCD subset contains a large number of frames, these frames are highly correlated within a driver and within a session and therefore cannot be regarded as independent samples. Consequently, the reported performance should be interpreted as evidence of the feasibility and within-driver/cross-session stability of the proposed transfer and fusion framework, rather than as evidence of generalizability across the driver population. The observed improvements may also be partially optimistic, since the test data share the same driver and acquisition conditions as the adaptation data. Establishing generalizability would require validation on a substantially larger cohort of independent drivers, across multiple vehicles, sessions, lighting conditions, and driving environments, which is identified as necessary future work.

The current evaluation focuses on validating the feasibility of the proposed transfer and fusion framework under a controlled data acquisition protocol. The robustness of the proposed method under illumination changes, large head pose variations, occlusions, and glasses has not been systematically evaluated, since the corresponding annotated data were not collected in this study. These factors are known to affect in-vehicle gaze estimation, and evaluating performance under such conditions would provide stronger support for practical applicability. Addressing these factors requires additional data collection under diverse lighting conditions, driving scenarios, and participant characteristics, which is identified as a primary direction for future work.

## 5. Conclusions

This study addresses the cross-scene adaptation problem in in-vehicle gaze estimation and constructs a unified framework consisting of the MIEF baseline model, composite multi-stage transfer learning paths (CMP1–CMP3), and a distributed multi-camera fusion model (MCF). The framework enables effective transfer adaptation of a gaze estimation model from a large-scale mobile-terminal source domain to a personalized in-vehicle multi-camera target scenario.

Experimental results demonstrate that composite multi-stage transfer learning effectively mitigates data distribution discrepancies between the source and target domains. In the cross-session evaluation, the CMP3 path achieves an average error of *μ* (*E*) = 0.277 across camera views and a best single-camera mean error of *μ* (*E*) = 0.244. Building on this, the distributed MCF model further exploits complementary information from multiple camera viewpoints: under the full C1 + C2 + C3 configuration, MCF-Plus achieves a mean error of *μ* (*E*) = 0.160, a standard deviation of *σ* (*E*) = 0.127, and 96.88% of predicted gaze points falling within the target calibration block.

Future research will focus on expanding the experimental scale to encompass a broader range of drivers, vehicle platforms, and complex driving scenarios. In particular, given that the present evaluation is based on only two drivers, validating the framework on a substantially larger cohort of independent drivers, across multiple vehicles, sessions, lighting conditions, and driving environments, is a necessary next step to establish generalizability. Further development of more lightweight and efficient multi-camera fusion architectures is also anticipated. In addition, online personalized adaptation strategies and the joint modeling of gaze heatmaps with driving-behavior semantics will be explored, with the goal of further enhancing model accuracy, robustness, and real-world applicability for personalized in-vehicle gaze estimation.

## Figures and Tables

**Table 1:** Experimental environment and training parameters.

CPU	GPU	RAM	Framework	Batch Size	Epochs	Optimizer	Momentum	Initial LR
Intel Core i9-10920X 3.50 GHz	NVIDIA TITAN RTX	16 GB	PyTorch	5	20	SGD	0.90	0.001

**Table 2:** Performance comparison of different training modes.

Stage	View	Mode	Source Pretraining	Transfer Learning	Evaluation	*μ* (*E*)	*σ* (*E*)	Approx. 95% CI of *μ* (*E*)	Error (*E*) <0.5 (%)	Error (*E*) >1.5 (%)
***Exp*.** 1	C1	DST	*𝒟_S_*	×	GCD1	7.023	2.920	[6.904, 7.142]	0.00	98.96
		TOT	×	GCD1	GCD1	1.952	2.245	[1.860, 2.044]	6.08	55.21
		STT	*𝒟_S_*	GCD1	GCD1	0.374	0.296	[0.362, 0.386]	80.56	1.74
	C2	DST	*𝒟_S_*	×	GCD1	6.456	3.403	[6.317, 6.595]	0.52	98.44
		TOT	×	GCD1	GCD1	1.878	2.102	[1.792, 1.964]	5.90	54.51
		STT	*𝒟_S_*	GCD1	GCD1	0.703	0.364	[0.688, 0.718]	31.94	3.47
	C3L	DST	*𝒟_S_*	×	GCD1	8.443	3.314	[8.308, 8.578]	0.00	99.83
		TOT	×	GCD1	GCD1	2.196	1.510	[2.134, 2.258]	4.86	66.49
		STT	*𝒟_S_*	GCD1	GCD1	0.471	0.810	[0.438, 0.504]	70.49	1.39
	C3R	DST	*𝒟_S_*	×	GCD1	8.400	3.293	[8.266, 8.534]	0.00	99.83
		TOT	×	GCD1	GCD1	2.195	2.353	[2.099, 2.291]	2.43	67.88
		STT	*𝒟_S_*	GCD1	GCD1	0.455	0.823	[0.421, 0.489]	73.78	1.04
***Exp*.** 2	C1	TOT	×	GCD2	GCD2	1.753	3.929	[1.593, 1.913]	30.21	14.93
		STT	*𝒟_S_*	GCD2	GCD2	0.302	0.242	[0.292, 0.312]	86.11	0.35
	C2	TOT	×	GCD2	GCD2	1.733	2.393	[1.635, 1.831]	11.98	38.37
		STT	*𝒟_S_*	GCD2	GCD2	0.436	0.234	[0.426, 0.446]	67.19	0.00
	C3L	TOT	×	GCD2	GCD2	1.702	1.995	[1.621, 1.783]	8.85	38.72
		STT	*𝒟_S_*	GCD2	GCD2	0.424	0.241	[0.414, 0.434]	69.97	0.00
	C3R	TOT	×	GCD2	GCD2	2.465	1.514	[2.403, 2.527]	5.56	69.62
		STT	*𝒟_S_*	GCD2	GCD2	0.339	0.213	[0.330, 0.348]	81.08	0.00
***Exp*.** 3	C1	CMP1	×	GCD2 → GCD3	GCD3	0.969	0.599	[0.945, 0.993]	17.19	11.46
		CMP2	*𝒟_S_*	GCD1 → GCD3	GCD3	0.313	0.161	[0.306, 0.320]	85.94	0.00
		CMP3	*𝒟_S_*	GCD2 → GCD3	GCD3	0.266	0.150	[0.260, 0.272]	91.49	0.00
	C2	CMP1	×	GCD2 → GCD3	GCD3	1.611	0.729	[1.581, 1.641]	12.52	33.87
		CMP2	*𝒟_S_*	GCD1 → GCD3	GCD3	0.364	0.199	[0.356, 0.372]	76.39	0.00
		CMP3	*𝒟_S_*	GCD2 → GCD3	GCD3	0.328	0.165	[0.321, 0.335]	86.28	0.00
	C3L	CMP1	×	GCD2 → GCD3	GCD3	1.271	1.033	[1.229, 1.313]	14.06	28.99
		CMP2	*𝒟_S_*	GCD1 → GCD3	GCD3	0.286	0.145	[0.280, 0.292]	92.36	0.00
		CMP3	*𝒟_S_*	GCD2 → GCD3	GCD3	0.268	0.143	[0.262, 0.274]	93.40	0.00
	C3R	CMP1	×	GCD2 → GCD3	GCD3	1.033	0.781	[1.001, 1.065]	14.58	14.24
		CMP2	*𝒟_S_*	GCD1 → GCD3	GCD3	0.254	0.156	[0.248, 0.260]	91.32	0.00
		CMP3	*𝒟_S_*	GCD2 → GCD3	GCD3	0.244	0.163	[0.237, 0.251]	95.49	0.00

Notes: Exp. 1, Exp. 2, and Exp. 3 refer to Experiment 1, Experiment 2, and Experiment 3, respectively.

**Table 3:** Performance comparison of multi-camera fusion.

Camera Configuration	Fusion Paradigm	*μ* (*E*)	*σ* (*E*)	Approx. 95% CI of *μ* (*E*)	Error (*E*) <0.5 (%)	Relative Error Reduction (%)
Baseline	Single-Camera Average	0.277	0.155	[0.271, 0.283]	91.67	—
C1 + C2	MCF-Lite	0.185	0.135	[0.179, 0.191]	95.31	33.2
	MCF-Plus	0.184	0.139	[0.178, 0.190]	95.14	33.6
C1 + C3	MCF-Lite	0.169	0.133	[0.164, 0.174]	96.70	39.0
	MCF-Plus	0.166	0.128	[0.161, 0.171]	96.53	40.1
C1 + C2 + C3	MCF-Lite	0.162	0.128	[0.157, 0.167]	96.70	41.5
	MCF-Plus	0.160	0.127	[0.155, 0.165]	96.88	42.2

## Data Availability

The source-domain pretraining dataset used in this study is the publicly available GazeCapture dataset [34]. The in-vehicle experimental datasets generated and analyzed during the current study are not publicly available due to privacy and institutional restrictions, but can be obtained from the corresponding author upon reasonable request.

## Abbreviations

The following abbreviations are used in this manuscript:

C1	Camera 1
C2	Camera 2
C3	Camera 3
C3L	Camera 3 left view
C3R	Camera 3 right view
CMP	Composite Multi-Stage Transfer Learning Path
DRD	Driving Gaze and Road Scene Data
DST	Direct Source Testing
FC^(L)^	Final fully connected layer
FC^(L−1)^	The output of the penultimate fully connected layer
GCD	Gaze Calibration Data
MCF	Multi-Camera Fusion
MIEF	Multi-Level Information Element Fusion
STT	Source-to-Target Transfer
TOT	Target-Only Training
*θ*_S_	Source-domain parameter set
*μ* (*E*)	Mean prediction error
*σ* (*E*)	Standard deviation of prediction errors
Ω_MF_^act^	Activated gaze meta-feature matrix
𝒟_S_	Source-domain pretraining dataset
𝒟_T_	Target-domain data collection
